# Signal Transduction Profiling of Angiotensin II Type 1 Receptor With Mutations Associated to Atrial Fibrillation in Humans

**DOI:** 10.3389/fphar.2020.600132

**Published:** 2020-12-22

**Authors:** Sarah C. Simões, André L. Balico-Silva, Lucas T. Parreiras-e-Silva, André L. B. Bitencourt, Michel Bouvier, Claudio M. Costa-Neto

**Affiliations:** ^1^ Ribeirao Preto Medical School, Department of Biochemistry and Immunology, University of São Paulo, Ribeirao Preto, Brazil; ^2^ Department of Biochemistry and Molecular Medicine and Institute for Research in Immunology and Cancer, University of Montréal, Montréal, QC, Canada

**Keywords:** Angiotensin II receptor, AngII, AT1 receptor, signalling, cardiovascular diseases, mutations

## Abstract

The AT1 receptor (AT1R) has a major role in the Renin-Angiotensin System, being involved in several physiological events including blood pressure control and electrolyte balance. The AT1R is a member of the G protein coupled receptors (GPCR) family, classically known to couple G_αq_ and engage β-arrestin recruitment. Both G protein and arrestin signaling pathways are involved in modulation of different downstream kinases. A previous study reported that mutations in the AT1R (A244S and I103T-A244S) were positively correlated with higher risk of atrial fibrillation in men. Based on that report, we aimed to investigate if these mutations, including I103T only, could affect AT1R signal transduction profile, and consequently, implicate in atrial fibrillation outcome. To address that, we engineered an AT1R carrying the above-mentioned mutations, and functionally evaluated different signaling pathways. Phosphokinase profiler array to assess the mutations downstream effects on kinases and kinase substrates phosphorylation levels was used. Our results show that the I103T-A244S mutant receptor presents decreased β-arrestin 2 recruitment, which could lead to a harmful condition of sustained G_αq_ signaling. Moreover, the phosphokinase profiler array revealed that the same mutation led to downstream modulation of kinase pathways that are linked to physiological responses such as fibrous tissue formation, apoptosis and cell proliferation.

## Introduction

G protein coupled receptors (GPCRs) represent the major family of cell surface receptors (Wise et al., 2004). It is estimated that about 40% of the marketed drugs have GPCRs as targets (Rask-Andersen et al., 2011; Hauser et al., 2017). The angiotensin II type 1 receptor (AT1R), a G protein coupled receptor, is the main receptor of the Renin-Angiotensin System, and angiotensin II (AngII) is the main agonist of this system. Along with the AT1R, AngII plays a direct role in the control of blood pressure, aldosterone release, electrolyte balance, and other physiological events (Fyhrquist and Saijonmaa, 2008). In addition, AT1R activation also promotes cell proliferation, inflammation and fibrosis (Forrester et al., 2018), which are related to cardiovascular diseases (de Gasparo et al., 2000). Activation of AT1R by AngII triggers the canonical G_αq_ protein signaling pathways, which leads to inositol trisphosphate (IP_3_) and diacylglycerol (DAG) generation, and Ca^2+^ mobilization from the endoplasmic reticulum, culminating in protein kinase C (PKC) and other downstream kinases activation, including ERK1/2. In addition, after AT1R activation by AngII, G protein coupled receptor kinases (GRKs) promote the phosphorylation of the receptor and β-arrestins recruitment, leading to receptor internalization and activation of other signaling pathways (Lima et al., 2014).

**FIGURE 1:**
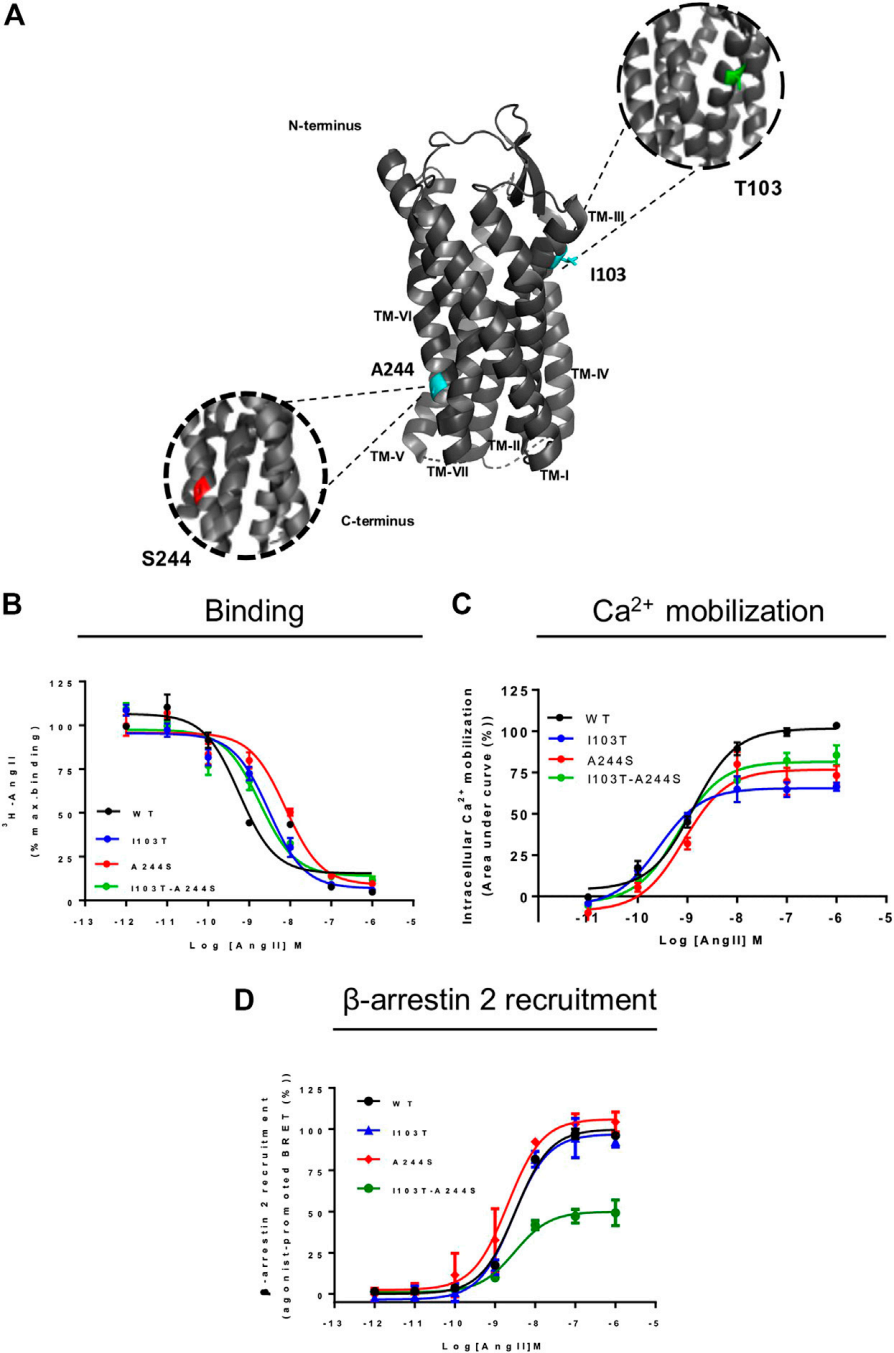
Position of the mutations correlated with atrial fibrillation and comparative analyses of signal profile between AT1 and mutant receptors. **(A)** Schematic representation of position of mutations in AT1 receptor. TM, transmembrane; cyan, original amino acids residues, Isoleucine and Alanine; green, Threonine substitution; red, Serine substitution. **(B)** Competition binding profiles for AngII against [^3^H]-AngII in HEK293T cells transiently expressing the AT1R and mutants. Data are expressed as percentages of the maximum specific binding of the radioligand. **(C)** Dose-response curve of the Ca^2+^ mobilization assay after AngII stimulation. Data are expressed as percentages of the maximum Ca^2+^ mobilization promoted by AT1R. **(D)** BRET assays showed that in the presence of AngII, AT1R and mutants promote β-arrestin 2 recruitment. Data are expressed as percentages related to the maximal recruitment promoted the AT1R. All data were generated from at least three independent experiments.

Anomalies in GPCRs caused by polymorphisms are associated with a variety of phenotypes and predisposition to certain diseases. It has been described more than 700 mutations that inactivate or over activate receptors, which are related to over 30 different human diseases (Schöneberg et al., 2004; Zalewska et al., 2014; Fukami et al., 2018). Marott, Nordestgaard et al. (2013) identified genetic variants of the AT1R in patients with atrial fibrillation (AF). The mutations A244S and I103T-A244S (Figure 1A) occur in conserved residues in mammals and were correlated with AF predisposition in men. It was reported that the risk of development of AF in heterozygotes for the variants A244S and I103T-A244S is higher when compared to individuals that do not carry these variants. It was then hypothesized that such mutations could possibly affect receptor responses, leading to an increase of inflammation, fibrosis, expression of gap junctions and ion channels, which could be related to the structural and electrical remodeling of the myocardial atrium (Marott et al., 2013).

Based on that, in this study we wanted to understand whether the A244S and I103T-A244S variants in the AT1R would affect the receptor signaling regarding its downstream pathway. We generated the A244S and I103T-A244S mutations in the human AT1R and characterized their impact in AngII affinity and signal transduction. We also evaluated the effects of the I103T mutant alone, which although not found and reported in the previous study, it was important here to allow comprehensive structure/function analyses in regard to the contributions of the mutation to the AT1R signaling profile and possible physiological effects.

## Materials and Methods

### Receptor Mutagenesis

The site-direct mutations in AT1 receptor: I103T, A244S and I103T-A244S were generated by whole plasmid PCR technique using oligonucleotide primers containing the A244S mutation (5′-GGC​AAT​TAC​CTA​TGT​AAG​ACT​GCT​TCA​GCC​AGC​GT 3’/5′-ACG​CTG​GCT​GAA​GCA​GTC​TTA​CAT​AGG​TAA​TT-3′) and the I103T mutation (5′-TTT​AAG​ATA​ATT​ATG​TCA​ATT​GTG​CTT​TTC​TTT-3’/5′-TTT​AAG​ATA​ATT​ATG​TCA​ATT​GTG​CT TTTCTTT-3′). Extension was performed using a high-fidelity DNA polymerase (Taq HiFi; Invitrogen). DpnI endonuclease (Invitrogen) was used to digest the parental DNA template. The mutants were sequenced with the Big Dye™ Terminator v3.1 Cycle Sequencing Ready Reaction Kit (Perkin Elmer) according to the manufacture’s instruction.

### Cell Culture and Transfections

HEK293T cells were cultivated in Dulbecco's Modified Eagle Medium from ThermoFisher Scientific company (DMEM) supplemented with 10% of fetal bovine serum (Vitrocell), 1 U/ml penicillin/streptomycin and incubated at 37°C in a 5% CO_2_ environment. Expression plasmids containing wild type (WT), mutated AT1 receptors and BRET-based biosensors were transiently transfected into cells using polyethylenimine (PEI 25 kDa linear; Polyscience) at a ratio of 3:1 PEI/DNA. The proportions of DNA of WT AT1R were adjusted according to a previous analysis of the expression level (see Supplementary Figure S1). When necessary, total DNA amount was adjusted with salmon sperm DNA (Invitrogen). Assays were performed 48 h after cell transfection.

### Competition Binding

HEK293T cells (3 × 10^5^ cells/well) transiently expressing the receptors were transferred to 24-well culture plates 24 h after the transfection. One day after plating, cells were washed once in cold buffer (25 mM Tris-HCl buffer, pH 7.4, containing 140 mM NaCl, 5 mM MgCl2 and 0.1% bovine serum albumin). Cells were incubated with a final concentration of 0.5 nM [^3^H]-angiotensin II and increasing concentrations of angiotensin II, as a competitor, in binding buffer (25 mM Tris-HCl, pH 7.4, including 5 mM MgCl2, 0.1% bovine serum albumin and 100 μg/ml bacitracin). Cells were maintained at 4°C for at least 16 h, washed twice and then lyzed with lysis buffer (48% urea, 2% Nonidet P-40, acetic acid 3M). Cell lysates were transferred to scintillation vials and 3 ml of scintillation liquid (UltimaGold™ XR; PerkinElmer) were added. Bound radioactivity was quantified on a Tri-Carb 20100 TR liquid scintillation counter (PerkinElmer).

### Intracellular Ca^2+^ Mobilization

Twenty-four hours after transfection, HEK293T cells transiently expressing the receptors were transferred to 96-well culture plates (ViewPlate-96; PerkinElmer), in a density of 5 × 10^4^ per well with 50 μl of DMEM without phenol supplemented with 10% FBS and 1U/ml penicillin/streptomycin. The cells were incubated after 48 h at 37°C with 50 μl of fluorescent dye indicator (FLIPR^®^ Calcium 5 Assay Kit; Molecular Devices) containing probenecid (Sigma), which can prevent the extrusion of the dye. FlexStation 3 Multi-Mode Microplate Reader (Molecular Devices) was used for the measurement of the emission at 525 nm after the excitation at 485 nm, after the injection of 25 μl of the agonist.

### β-arrestin 2 Recruitment

HEK293T cells (4 × 10^4^/well) transiently transfected with mutant receptors (1 µg) or WT AT1R (500 ng), βarrestin2-RlucII (25 ng), rGFP-CaaxBox (400 ng) and salmon sperm to complete 2 µg of total DNA, were seeded in white opaque 96-well microplates (OptiPlate; PerkinElmer). Forty-eight hours after transfection, bioluminescence resonance energy transfer 2 (BRET2) was monitored in a VictorX Light Luminescence Microplate Reader (PerkinElmer) equipped with different donor/acceptor emission filter sets after 15 min of AngII addition, and 5 min after 2.5 mM of coelenterazine 400-a addition (Biotium) to the cells. BRET2 signals were derived from the ratio between the emission detected with the energy acceptor filter (515 ± 20 nm) and the emission detected using the energy donor filter (400 ± 70 nm). βarrestin2-RlucII and rGFP-CaaX expression plasmids were generated at the laboratory of Dr. Michel Bouvier at the University of Montreal, Canada (Parreiras-E-Silva et al., 2017; Namkung et al., 2018).

### Phospho-Kinase Profiler Array

The Human Phospho-Kinase Antibody Array (R&D Systems) was used as described previously (Santos et al., 2015). Briefly, HEK293T cells (6 × 10^6^ cells/well) were seeded on 10 cm^2^ culture plates and transfected with the WT receptor (2.5 µg), mutant receptors (5 µg), and salmon sperm to complete a total DNA amount of 10 µg. After 24 h, cells were serum starved for 16 h and then stimulated at 37°C with 100 nM of AngII as the final concentration for 10 min. Cells were rinsed, lyzed, and the homogenate was clarified by centrifugation at 14,000 × *g* for 5 min at 4°C. A sample of 300 µg of total protein from the lysates was then incubated with the pre-blotted membrane array. After the incubation period, ECL™ prime (GE Healthcare) was added and the chemiluminescent signal was captured by ImageQuant 350 (GE Healthcare). For phosphorylation levels analysis, membranes were scanned, and blots were quantified using Image J software. The intensities of the spots were quantified and divided by the values obtained for the WT AT1R (see Supplementary Figure S2; Supplementary Table S1). Substrate’s phosphorylation levels were considered differentially modulated when values reached 30% above or below, as compared with the values obtained for the WT AT1R.

### ERK1/2 Phosphorylation

Twenty-four hours after transfection, 3 × 10^5^ cells were seeded on 6-well plates in DMEM supplemented with 10% fetal bovine serum, 1 U/ml penicillin/streptomycin, in a 37°C and 5% CO_2_ environment. After 24 h, cells were serum starved for 16 h and then stimulated with 100 nM AngII as the final concentration for 5, 10, 15, 20, 25 or 30 min at 37°C, and analyzed for ERK phosphorylation. Cells were lyzed with cold lysis buffer (Tris-HCl 10 mM, pH 7.5, NaCl 150 mM, EDTA 1 mM, EGTA 1 mM, SDS 0.1%, Nonidet P-40 1%, SIGMAFAST™ protease inhibitor cocktail, sodium orthovanadate 1 mM and sodium fluoride 10 mM). Following homogenization during 30 min at 4°C, cell lysate was centrifuged for 15 min at 4°C and 13,000 rpm. Total protein was quantified using Bradford protein assay. Subsequently, 60 µg of total protein were separated in SDS-PAGE 12%, transferred to nitrocellulose membrane and western blotting was performed against total ERK2 and phosphorylated ERK1/2 (both antibodies from Santa Cruz Biotechnology catalog number: sc-1647 and cs-377400 respectively). Densitometry of the bands were analyzed using ImageJ program (http://rsb.info.nhi.gov/ij/) and the ratio phosphorylated ERK/total ERK was used to access ERK1/2 activation. Corresponding results were plotted using GraphPad Prism 7 software as the percentage of the maximum ratio values (GraphPad).

### Statistical Analyses

Data are expressed as mean ± standard error of the mean (S.E.M.) from at least three independent experiments. Sigmoid curves from concentration-response experiments were analyzed using nonlinear curve fitting. Binding, Ca^2+^ mobilization and β-arrestin 2 recruitment data were analyzed by one-way Analysis of Variance (ANOVA). When appropriate, Dunnett’s *post hoc* test was used. Significance level was set at *p* < 0.05. Statistical analyses were carried out using GraphPad Prism 7 (GraphPad) software and are indicated in the legends of the figures.

## Results

### Evaluation of AngII Affinity by the Mutant Receptors

AngII affinities for the mutant receptors were assessed by competition binding assay using radiolabeled ^3^H-AngII, and non-radioactive AngII in different concentrations as the competitor ligand. Considering that the receptors containing the mutations I103T or A244S, as well as the combination of both mutations, are 50% less expressed in relation to the WT receptor, the transfection levels were adjusted for all assays (see Supplementary Figure S1). As it can be seen in Figure 1B, the competition binding profiles revealed that AngII, in general, has a slightly lower affinity for the mutant receptors, as compared with the WT receptor. Table 1 describes the obtained affinities for AngII in the mutant and WT receptors. It is noteworthy to observe that the A244S mutation caused a ∼10-fold decrease in AngII affinity, and that the insertion of the second mutation (i.e. generation of the double mutant I103T-A244S) resulted in the recovery of the high affinity profile and the partial recovery of the receptor’s capacity to lead to calcium mobilization.

**TABLE 1:** Binding affinity, potency and relative efficacy (E_max_) of AngII to promote Ca^2+^ mobilization and β-arrestin 2 recruitment.

Receptor	Binding			Ca^2+^ Mobilization			β-arrestin 2 recruitment		
	*p*IC_50_	*n*	K_d_ (nM)	*p*EC_50_	*n*	E_max_ (% WT)	*p*EC_50_	*n*	E_max_ (% WT)
WT	9.00 ± 0.17	3	0.63 ± 0.51	8.89 ± 0.08	5	100	8.49 ± 0.05	3	100
I103T	8.63 ± 0.19	3	1.18 ± 0.94	9.57 ± 0.13*	3	65 ± 4	8.54 ± 0.08	3	97 ± 2
A244S	8.19 ± 0.12*	3	6.33 ± 2.0	9.07 ± 0.17	5	85 ± 15	8.68 ± 0.10	3	106 ± 2
I103T-A244S	8.61 ± 0.10	3	2.15 ± 0.67	9.17 ± 0.09	3	81 ± 5	8.50 ± 0.10	3	50 ± 4

HEK293T expressing AT1R/mutant receptors were stimulated with various concentrations of AngII. Ang II binding affinities were obtained from [^3^H]-AngII competition binding assay. Ca^2+^ mobilization and BRET were normalized to the maximal response of WT (% E_max_ of WT) and then averaged. pEC_50_ and E_max_ were obtained from the nonlinear regression curve of the averaged data. Data represents the means ± SEM of three or more independent experiments. *p < 0.05 compared to the WT receptor.

### Analysis of Mutant Receptors’ Signaling Transduction Pathways

We evaluated whether the different mutations could affect Ca^2+^ mobilization and β-arrestin 2 recruitment after AngII stimulation, two well-known signaling pathways for the AT1R (Chang et al., 1992; Wu et al., 1992). The results show that in Ca^2+^ mobilization assays all mutant receptors yielded potencies similar to the WT AT1R, and moderate lower efficacies (Figure 1C). Concerning β-arrestin 2 recruitment, the I103T-A244S mutation yielded a profound decrease in the translocation of β-arrestin 2 to membrane (reduction of ∼50%), while the I103T and A244S mutants presented profiles similar to the WT AT1R (Figure 1D).

### Profiling of Kinases Pathways Modulated by Mutant Receptors

**FIGURE 2:**
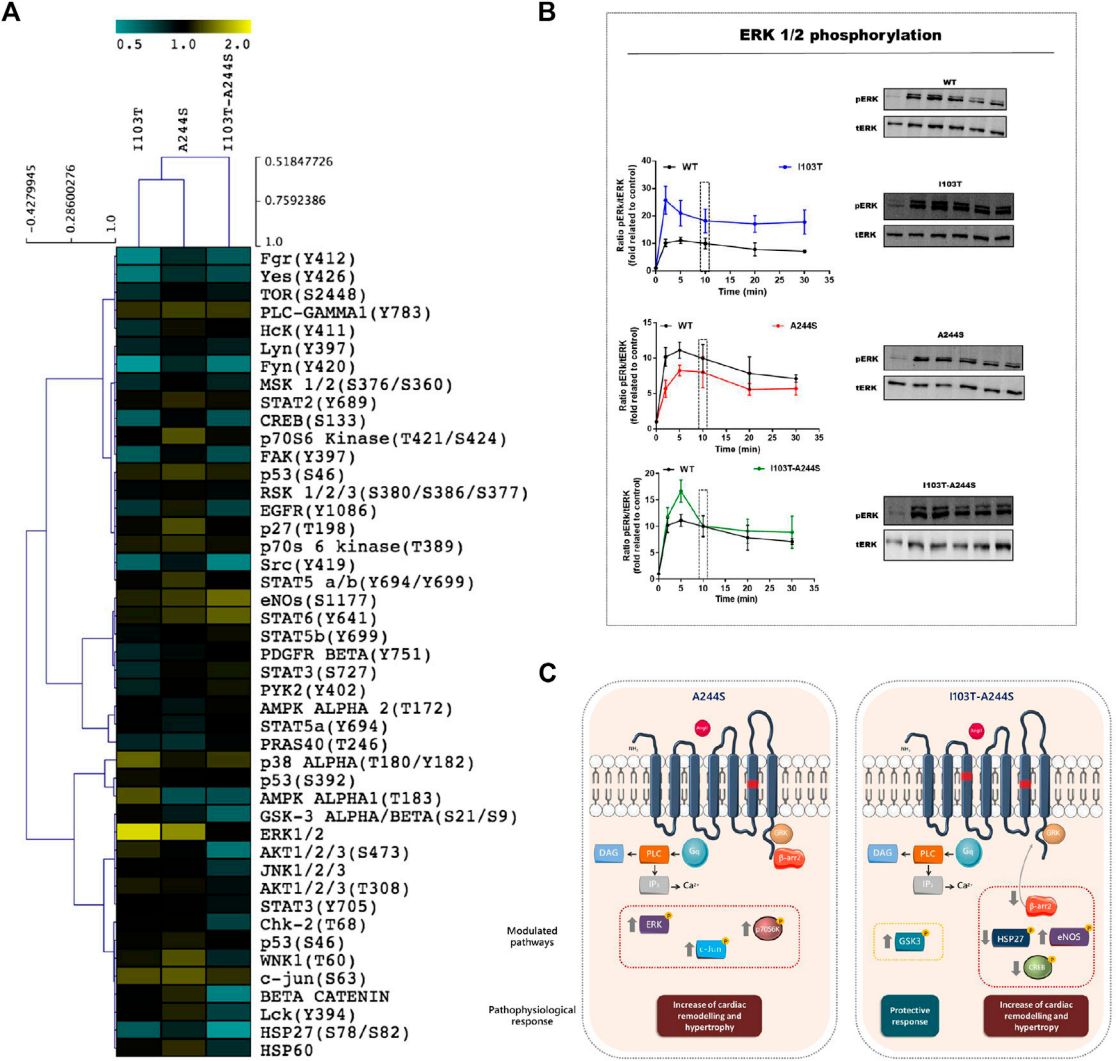
Modulation profile of kinases after stimulation of the receptors with the endogenous agonist and signaling pathways affected by the A244S and I103T-A244S mutants. **(A)** HEK293T cells transiently expressing the AT1R and mutant receptors were incubated with 100 nM of AngII for 10 min. The heatmap was generated using the ratio between spots quantified in mutant receptors membrane/spots quantified in WT membrane. **(B)** Kinetics of ERK1/2 phosphorylation after stimulation with 100 nM AngII (at least 3 independent experiments). **(C)** Gαq and β-arrestin interaction with AT1 mutant receptors is shown, and the kinases that were up- or down-regulated, as well as the alteration in β-arrestin 2 recruitment, are highlighted. The physiological consequences due to the modulation of each cluster of proteins are indicated. Up arrows indicate up-modulation, and down arrows represent down-modulation.

The phosphorylation patterns of different kinase substrates were analyzed from lysates of AngII-stimulated HEK293T cells transfected with each of the mutant receptors or the WT AT1R. The membrane blots were quantified and values plotted as a heat-map (Figures 2A; Supplementary Figure S2). Values of modulation fold can be found in Table 2 (see also Supplementary Table S1 for a list of all kinase substrates included in the array and their modulation fold).

**TABLE 2:** Kinases modulated by the mutant receptors in comparison to the WT after 10 min of stimulus with AngII.

Modulated proteins	Fold change (spots quantified in mutant receptor membrane/spots quantified in WT membrane)		
	I103T	A244S	I103T-A244S
p38α	1.39 ▲	1.10	1.23
ERK1/2	1.85 ▲	1.55 ▲	1.03
GSK-3α/β	1.05	0.90	0.67 ▼
AMPKα1	1.32 ▲	0.73	0.73
AKT1/2/3 (S473)	1.17	0.96	0.62 ▼
CREB	0.69 ▼	0.97	0.70 ▼
HSP27	0.70 ▼	0.87	0.46 ▼
β-Catenin	0.98	1.17	0.58 ▼
c-jun	1.31 ▲	1.37 ▲	1.20
Src	0.67 ▼	0.90	0.57 ▼
p70 S6 kinase	1.03	1.32 ▲	1.06
eNOs	1.16	1.24	1.42 ▲
Fyn	0.48 ▼	0.85	0.60 ▼
Yes	0.60 ▼	0.84	0.74
Fgr	0.56 ▼	0.84	0.70 ▼
STAT6	1.11	1.22	1.37 ▲
p27	1.06	1.30 ▲	1.03
WNK1	1.10	1.33 ▲	0.86

HEK293T protein cell lysates expressing wild-type AT1R or mutant receptors were stimulated with Ang II and incubated with the phosphokinase array membranes. Results were plotted using the ratio between spots quantified in mutant receptors membrane/spots quantified in WT receptor membrane. Only kinases or substrates with intensity changes of ±30% were considered to be affected by the condition (indicated by a black triangle. ▲: up-modulation ▼: down-modulation).

We identified 18 kinases and kinases substrates which phosphorylation levels were modulated more than 30%, by at least one of the three mutant receptors, when compared to WT receptor. The A244S mutant receptor led to increased phosphorylation levels of kinases such as ERK1/2, c-Jun and p70 S6 kinase. For the I103T mutant, ERK1/2 MAP kinases showed an increased phosphorylation. The activation of the I103T-A244S double mutant resulted in the decreased phosphorylation levels of cyclic AMP response element binding protein (CREB) and of glycogen synthase kinase-3 α/β (GSK3 α/β), but also led to the elevation of endothelial nitric oxide synthase (eNOS) phosphorylation levels. The HSP27 presented a lower phosphorylation profile for all the mutant receptors, but it was more pronounced for the I103T-A244S mutant.

### Analysis ERK1/2 Phosphorylation Kinetics

The ERK1/2 MA P kinase activation was also assessed by western blot after AngII stimulation for different times (Figure 2B). We found that the I103T mutation led to ∼3-fold increased ERK1/2 phosphorylation levels, while both A244S and I103T-A244S mutants presented phosphorylation profiles similar to the WT AT1R. The peak of ERK 1/2 activation at 5 min is 1.5-fold higher after I103T-A244S mutant stimulation as compared to the WT receptor. These results corroborate the modulation levels observed in the phospho-kinases activation profile (see Figure 2A).

## Discussion

The AT1R is directly involved in the establishment and progression of cardiovascular diseases, such as hypertension, heart failure, among others (Hunyady and Catt, 2006). Marott et al. (2013) based on a genetic study with patients with AF, reported that I103T-A244S and A244S mutations in the AT1R were directly associated with the predisposition to the development of this condition in men. Indeed, the I103T and A244S mutations (Figure 1A) are located respectively in the AT1R transmembranes helices 3 (TM3) and 6 (TM6), regions that have been described to bear critical residues that compose binding pockets for peptide and non-peptide ligands in different GPCRs (de Gasparo et al., 2000; Martin et al., 2004; Balakumar and Jagadeesh, 2014).

Thus, in the present study we aimed to evaluate whether the presence of the I103T-A244S and A244S would affect the downstream signaling of the AT1R, which in turn could have possible implications in AF. To allow a full structural/functional analysis, and as well to better understand why only A244S and the double mutation have been correlated with AF, we also generated and analyzed the I103T AT1R mutant. Our data show the decrease in β-arrestin recruitment by I103T-A244S mutant, which could be related to distinct conformations stabilized by the mutant receptor-ligand complex disfavoring arrestin coupling, since calcium mobilization was not affected in a large extent. Considering the impaired I103T-A244S mutant receptor interaction with β-arrestin, we inferred that this receptor should present a lesser extent of internalization. Based on that, a possible correlation with the clinical outcome is that the damaging effects of G_αq_ sustained activation, such as cardiomyocytes apoptosis, vasoconstriction and cardiac hypertrophy could be prolonged, impairing the AT1R receptor signaling and eventually contributing to AF development (Adams et al., 1998; Adams et al., 2000). Considering the impaired I103T-A244S mutant receptor interaction with β-arrestin, we inferred that this receptor should present a lesser extent of internalization. Based on that, a possible correlation with the clinical outcome is that the damaging effects of G_αq_ sustained activation, such as cardiomyocytes apoptosis, vasoconstriction and cardiac hypertrophy, could eventually favoring AF development (Adams et al., 1998; Adams et al., 2000). It is interesting to highlight that the receptor bearing only the I103T mutation shows an unaffected recruitment of β-arrestin as compared to the WT receptor.

Besides the activation of direct effectors (i.e. G protein and arrestins), AT1R signaling also promotes downstream phosphorylation of different kinases and substrates that are involved in alterations in extracellular matrix, gap junction formation, ion channels functionality, and others (DeWire et al., 2007; Shaul and Seger, 2007; Forrester et al., 2018). Mutations may change the conformation of a receptor. This change may induce different signaling responses after the activation with agonists or the inactivation with antagonists. The ligand’s induced change in the mutant receptor’s conformation may or may not favor its interaction with other signaling molecules (Wingler et al., 2019). Many of those signaling molecules are the kinases, which control a myriad of processes, such as apoptosis and cell proliferation, key events related to atrial remodeling, development and progression of AF (Zheng et al., 2014; Zheng et al., 2015; Nattel, 2017). In the current study, 18 kinases and kinases’ substrates phosphorylation levels suffered modulation of at least 30% by at least one of the three mutant receptors, when comparted to WT AT1R. Interestingly, some of the identified kinase substrates have been described to be involved in physiological processes that are associated to the development of AF.

ERK1/2, c-Jun and p70 S6 kinase, which have been related to the predisposition to cardiac injuries (for review, see Forrester et al., (2018)), had their phosphorylation levels increased by the A244S. In fact, studies show that ERK1/2, when upregulated, stimulates fibrotic tissue formation in heart. This condition contributes to cardiac hypertrophy and AF development (Shaul and Seger, 2007; Mebratu and Tesfaigzi, 2009; Wang et al., 2015). The I103T mutation is apparently the triggering-mechanism for increasing the receptor's ability to activate the ERK1/2. In the double mutant, the A244S mutation promotes the balance of this signaling, since it reduces the ability of the receptor to promote activation of ERK1/2, reaching levels that are closer to the WT receptor. The reduction of the phosphorylation level of CREB induced by the I103T-A244S mutant can also be associated to the AF phenotype, and the results indicate that the mutation responsible for this event is I103T. As a previous study showed that a decreased expression of genes modulated by CREB are related to atrial remodeling, alterations in metabolism, impairment of the cardiac contractility function and regulation of the electric activity (Mayr and Montminy, 2001; Seidl et al., 2017). In addition to this, the elevation of eNOS phosphorylation levels indicates that the double mutant receptor may be involved in the predisposition to AF condition, as eNOS activation has a role in endothelial dysfunction and in the blood pressure increase (Daiber et al., 2019).

An important protein with a cardioprotective role is the HSP27 (Wu et al., 2015), which presented a specially lower activation for the I103T-A244S double mutant, indicating the possible contribution of such polymorphism to the progression of the disease. Glycogen synthase kinase-3 α/β (GSK3 α/β) is another key protein that presented modulated phosphorylation levels in I103T-A244S transfected cells. It is described that the isoforms α and β of GSK3 are active when dephosphorylated, acting against the growth of cardiac cells and protecting fibrosis tissue formation (Lal et al., 2015). Therefore, the decreased phosphorylation levels observed for GSK3 suggest an increased activity of both isoforms, which in turn, might be considered beneficial.

Besides the above-mentioned targets, which were modulated over threshold, a modest modulation was also observed for other substrates (Figure 2A). For example, WNK isoforms, Src and Src-related kinases, AMPK, GSK3/Akt pathway, among others, showed a different phosphorylation profile induced by the analyzed mutations, when compared to WT AT1R. All of them have been described to be involved on different cardiovascular dysfunctions, fibrosis and atrial fibrillation (Penela et al., 2001; Fessart et al., 2005; Kim et al., 2005; Gavi et al., 2006; Kraja et al., 2011; Harada et al., 2015; Qi et al., 2017; Van Gastel et al., 2018), thus these signaling pathways could also be contribute to the AF phenotype induced by the studied AT1 mutations.

Our results reveal that the AF-related AT1R mutants have their signaling profiles altered, as illustrated in Figure 2C. For instance, when stimulated with AngII the A244S mutant led to phosphorylation of kinases that are involved in aggravating AF. Also, our data suggest that the AF condition may be more critical in patients that carry the AT1R I103T-A244S variants, as recruitment of β-arrestin 2 was significantly reduced. Furthermore, kinase substrate profiling after activation of the double mutant receptor suggests that it can be related to higher predisposition of AF development.

Moreover, considering that the mutations led to a reduced receptor expression in relation to the WT receptor, the effects in the individuals bearing such mutations may be due to the expression *per se*, or also a consequence of the alteration in the receptor capacity to form homodimers and heterodimers, which may interfere in the AT1R signaling profile. It is also important to mention that the physiological impact of these mutations needs to be addressed in future studies using animal models of atrial fibrillation. Also, in the context of biased agonism of GPCR, which has been envisioned as a promise for better or more specific therapies, analysis of different AT1R ligands for treatment of AF shall also be studied.

## Data Availability Statement

The raw data supporting the conclusions of this article will be made available by the authors, without undue reservation.

## Author Contributions

SS conceived the research. SS and AS carried out the experiments and data analysis. AB processed the experimental data of the phospho-kinase profiler array. SS and AS wrote the manuscript with support from LP-S, AB, CC-N, and MB. LP-S and CC-N supervised the project.

## Funding

This research was supported by the Sao Paulo State Research Foundation (FAPESP), Grant 2012/20148-0 to CC-N, and by Grants from the Canadian Institute for Health Research to MB. CC-N and MB had a joint international cooperation grant funded by FAPESP, Grant SPRINT 2015/50086-4. LP-S holds a Young Researcher grant from FAPESP (2016/24120-3). This study was financed in part by the Coordination for the Improvement of Higher Education Personnel (CAPES - Brazil) - Finance Code 001. SCS was recipient of a FAPESP fellowship (2016/08920-0). ALBS was recipient of a CAPES fellowship (88882.179973/2018-01).

## Conflict of Interest

The authors declare that the research was conducted in the absence of any commercial or financial relationships that could be construed as a potential conflict of interest.
