# Supplementary material for: Signal Transduction Profiling of Angiotensin II Type 1 Receptor With Mutations Associated to Atrial Fibrillation in Humans
Source: Front Pharmacol. 2020 Dec 22;11:600132. doi: 10.3389/fphar.2020.600132 (PMC7786401; doi:10.3389/fphar.2020.600132)
Supplement: Supplementary file 1 [file datasheet1.docx]

Supplementary Material

## Supplementary Figures

Binding

**Supplementary Figure 1. Competition binding assay profile.**  HEK293T cells transiently expressing the AT1R and mutants were incubated with AngII in two different concentrations (10^-12^ M and 10^-6^ M), and [^3^H]-AngII (5x10^-10^ M) was used as radioligand. Different DNA amounts of the plasmid containing WT-AT1R were used to evaluate the specific binding, which can be correlated with the number of receptors per cell. Total DNA amount for transfection was 10 µg and, when the coding plasmids did not reach such amount, it was adjusted using salmon sperm DNA. Data are expressed as percentages of the maximum specific binding of the radioligand, and were generated from at least 3 independent experiments.


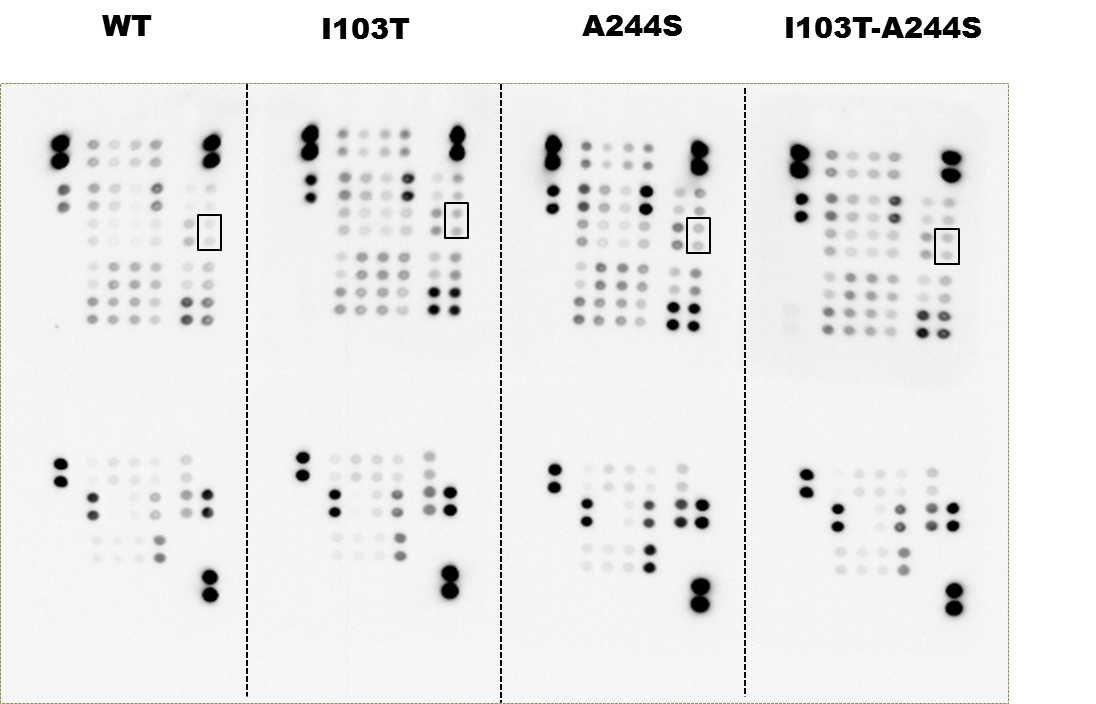


**Supplementary Figure 2. Membranes of the phosphokinase array.**  ERK phosphorylation spots are indicated by a black rectangle.

**Supplementary Tables**

**Supplementary Table 1. Complete modulation kinase profiles promoted by receptors stimulus with AngII for 10 min.** Cell lysates of HEK293T expressing WT or mutant AT1 receptors and stimulated with AngII were incubated with the membranes of the phosphokinase array profiler. Results were plotted using the ratio between the spots quantified in mutant receptors membrane and the spots quantified in WT receptor membrane.

| **Membrane** | **Column** | **Line** | **Proteins** | **Fold Change** | | |
| --- | --- | --- | --- | --- | --- | --- |
|  |  |  |  | **I103T** | **A244S** | **I103T-A244S** |
| **A** | **A** | **1.2** | **reference spot** | 1.10 | 1.22 | 1.52 |
|  |  | **3.4** | **p38α** | 1.39 | 1.10 | 1.23 |
|  |  | **5.6** | **ERK1/2** | 1.85 | 1.55 | 1.03 |
|  |  | **7.8** | **JNK1/2/3** | 1.03 | 0.98 | 0.82 |
|  |  | **9.10** | **GSK-3α/β** | 1.05 | 0.90 | 0.67 |
| **B** |  | **13.14** | **p53 (S392)** | 1.08 | 1.02 | 0.99 |
|  |  | **17.18** | **reference spot** | 1.18 | 1.07 | 1.09 |
| **A** | **B** | **3.4** | **EGFR** | 0.81 | 1.12 | 0.78 |
|  |  | **5.6** | **MSK 1/2** | 0.85 | 1.02 | 0.88 |
|  |  | **7.8** | **AMPKα1** | 1.32 | 0.73 | 0.73 |
|  |  | **9.10** | **AKT1/2/3 (S473)** | 1.17 | 0.96 | 0.62 |
| **B** |  | **11.12** | **AKT1/2/3 (T308)** | 1.12 | 1.08 | 0.93 |
|  |  | **13.14** | **p53 (S46)** | 1.15 | 1.25 | 1.14 |
| **A** | **C** | **1.2** | **TOR** | 0.83 | 0.98 | 0.91 |
|  |  | **3.4** | **CREB** | 0.69 | 0.97 | 0.70 |
|  |  | **5.6** | **HSP27** | 0.70 | 0.87 | 0.46 |
|  |  | **7.8** | **AMPKα2** | 1.01 | 0.91 | 1.04 |
|  |  | **9.10** | **β-Catenin** | 0.98 | 1.17 | 0.58 |
| **B** |  | **11.12** | **p70s 6 kinase** | 1.12 | 1.20 | 1.08 |
|  |  | **13.14** | **p53 (S15)** | 1.05 | 1.12 | 0.98 |
|  |  | **15.16** | **c-jun** | 1.31 | 1.37 | 1.20 |
| **A** | **D** | **1.2** | **Src** | 0.67 | 0.90 | 0.57 |
|  |  | **3.4** | **Lyn** | 0.86 | 0.94 | 0.88 |
|  |  | **5.6** | **Lck** | 1.03 | 1.16 | 0.77 |
|  |  | **7.8** | **STAT2** | 1.06 | 1.17 | 1.08 |
|  |  | **9.10** | **STAT5a** | 0.98 | 0.91 | 1.01 |
| **B** |  | **11.12** | **p70S6 Kinase** | 1.03 | 1.32 | 1.06 |
|  |  | **13.14** | **RSK 1/2/3** | 0.95 | 1.04 | 0.94 |
|  |  | **15.16** | **eNOs** | 1.16 | 1.24 | 1.42 |
| **A** | **E** | **1.2** | **Fyn** | 0.48 | 0.85 | 0.60 |
|  |  | **3.4** | **Yes** | 0.60 | 0.84 | 0.74 |
|  |  | **5.6** | **Fgr** | 0.56 | 0.84 | 0.70 |
|  |  | **7.8** | **STAT6** | 1.11 | 1.22 | 1.37 |
|  |  | **9.10** | **STAT5b** | 0.94 | 1.00 | 1.08 |
| **B** |  | **11.12** | **STAT-3 (Y705)** | 1.01 | 0.98 | 0.93 |
|  |  | **13.14** | **p27** | 1.06 | 1.30 | 1.03 |
|  |  | **15.16** | **PLC-γ1** | 1.20 | 1.26 | 1.23 |
| **A** | **F** | **1.2** | **HcK** | 0.83 | 1.09 | 1.02 |
|  |  | **3.4** | **Chk-2** | 0.98 | 1.02 | 0.78 |
|  |  | **5.6** | **FAK** | 0.71 | 0.95 | 0.74 |
|  |  | **7.8** | **PDGF Rβ** | 0.86 | 0.94 | 0.99 |
|  |  | **9.10** | **STAT5 a/b** | 1.05 | 1.22 | 1.00 |
| **B** |  | **11.12** | **STAT-3 (S727)** | 0.85 | 1.05 | 1.11 |
|  |  | **13.14** | **WNK1** | 1.10 | 1.33 | 0.86 |
|  |  | **15.16** | **PYK2** | 0.87 | 1.03 | 1.10 |
| **A** | **G** | **1.2** | **reference spot** | 0.85 | 1.26 | 1.42 |
|  |  | **3.4** | **PRAS40** | 0.86 | 0.82 | 0.96 |
|  |  | **9.10** | **PBS** | 0.00 | 0.00 | 0.00 |
| **B** |  | **11.12** | **HSP60** | 1.05 | 1.18 | 0.84 |
|  |  | **17.18** | **PBS** | 0.00 | 0.00 | 0.00 |
